# An Idle Rumour Resting on Malice

**Published:** 1917-12-22

**Authors:** Arthur Stanley

**Affiliations:** Chairman of the Council. The College of Nursing, Limited, 6 Vere Street, W. 1.


### THE EDITOR'S LETTER-BOX.

### An Idle Rumour Resting on Malice.

*To the Editor of* The Hospital.

Sir,—With reference to a report being constantly circulated that the V.A.D. Nursing Member is to be admitted to the membership of the College of Nursing, I shall be obliged if you will make it known that the College stands exclusively for the trained nurse.

V.A.D. Nursing Members who wish to become members of the College must serve their full training and hold a certificate of a recognised general training-school.—I am, Sir, yours faithfully,

(Signed) Arthur Stanley,

Chairman of the Council.

The College of Nursing, Limited,
6 Vere Street, W. 1.
December 19, 1917.

